# Variant analysis in Chinese families with hereditary hemorrhagic telangiectasia

**DOI:** 10.1002/mgg3.893

**Published:** 2019-08-10

**Authors:** Yali Zhao, Yuan Zhang, Xiangdong Wang, Luo Zhang

**Affiliations:** ^1^ Department of Otolaryngology Head and Neck Surgery Beijing TongRen Hospital, Capital Medical University Beijing China; ^2^ Beijing Key Laboratory of Nasal Diseases Beijing Institute of Otolaryngology Beijing China; ^3^ Department of Allergy Beijing TongRen Hospital, Capital Medical University Beijing China

**Keywords:** *ACVRL1*, *ENG*, epistaxis, hereditary hemorrhagic telangiectasia, variants

## Abstract

**Background:**

Hereditary hemorrhagic telangiectasia (HHT) is a vascular dysplasia disorder characterized by epistaxis, mucocutaneous telangiectasias and arteriovenous malformations in internal organs. Recurrent epistaxis is the primary complaint in 90%‐96% of HHT patients and the other symptoms come with age. The aim of this study was to analyze HHT‐associated gene variant spectrum in Chinese HHT patients and to assess whether genetic testing could contribute to the early diagnosis.

**Methodology/Principal:**

Thirty one HHT families including 62 individuals were recruited. Variants in the coding regions of four genes involved in HHT were amplified and analyzed using Sanger sequencing and multiplex ligation‐dependent probe amplification (MLPA).

**Results:**

Twenty unique variants, including 8 novel variants were found in 24 of the 31 (77.4%) kindred. Diagnosis is confirmed for 7 possible individuals from 6 kindred. Thirteen *ACVRL1* variants were detected from 17 isolated HHT families. Variants in *ACVRL1* from 8/17 (47.1%) families were located in exon8. Seven *ENG* variants were found in 7 unrelated families throughout the coding region.

**Conclusion:**

We conclude that *ACVRL1* gene variant is 2.4 times more prevalent than that in *ENG* in Chinese individuals with HHT, and exon8 of the *ACVRL1* gene may be a hotspot region. Genetic testing could contribute to early diagnosis for HHT.

## 1 INTRODUCTION

Hereditary hemorrhagic telangiectasia, also known as Rendu‐Osler‐Weber syndrome, is a rare autosomal dominant genetic disorder, which affects 1 in 5–8,000 individuals (Faughnan et al., 2011; Govani & Shovlin, 2009; Lesca et al., 2007; Shovlin, 2010). Characteristic features of HHT include recurrent epistaxis, the presence of mucocutaneous telangiectasias, arteriovenous malformations (AVMs) in internal organs, and family history of HHT (Guttmacher, Marchuk, & White, 1995). The clinical diagnosis of HHT is based on the Curacao criteria (Shovlin et al., 2000), which propose that three or more of the four characteristic features described above define a definite diagnosis, where as two of these features suggest a “possible” diagnosis and one or none of these features indicate unlikely HHT. The penetrance for HHT is age‐dependent. Epistaxis is the first and the primary manifestation in 90%–96% of HHT patients(Guttmacher et al., 1995). Thus, the diagnosis for children and sporadic patients with recurrent epistaxis only is hard to decide.

At least four genes, including Endoglin (*ENG,* OMIM: 131195)(McAllister et al., 1994) resulting in HHT1 (OMIM: 187300), Activin A Receptor Type II‐like 1 (*ACVRL1,* OMIM: 601284) resulting in HHT2 (OMIM: 600376) (Johnson et al., 1996), SMAD family member 4(*SMAD4,* OMIM: 600993) resulting in HHT syndrome associated with juvenile polyposis (JP‐HHT, OMIM: 175050) (Gallione et al., 2006) and Bone morphogenetic proteins 9(*BMP9,* OMIM: 605120) resulting in a vascular anomaly syndrome (HHT5, OMIM: 615506) (Wooderchak‐Donahue et al., 2013), are thought to be responsible for about 90% HHT patients diagnosed by the clinical features. The remaining ∼10% of HHT patients have an unidentified genetic cause, which may be resulted from intronic variants in the known genes or caused by a novel gene (McDonald et al., 2011; Wooderchak‐Donahue et al., 2018). The aforementioned genes were all part of the transforming growth factor (TGFβ) signal pathway and integral to angiogenesis. Pathogenic variants in any of these genes may disrupt the balance between pro‐ and antiangiogenic signals for normal vascular development, resulting in HHT.

Previous studies have indicated that the disorder was caused predominantly by variants in either *ENG* (McAllister et al., 1994; McDonald et al., 1994; Shovlin et al., 1994) or *ACVRL1* (Johnson et al., 1995; Vincent et al., 1995) genes. More than 500 variants have been reported in the two genes. Many of the variants were specific for each family, however, recurrent or founder variant has been reported in some populations, suggesting that the variant spectrum for HHT families may vary in different populations. Indeed, it has been shown that American, North European and Japanese families have fewer *ACVRL1* variants than *ENG* variants (Komiyama, Ishiguro, Yamada, Morisaki, & Morisaki, 2014; McDonald et al., 2011). Presently there is only one report in the literature on the clinical and genetic characteristics of Chinese HHT patients (Chen et al., 2013). Thus, the aim of our study was to expand on this database on the variant spectrum of Chinese patients with HHT, and to assess whether genetic testing could set the diagnosis for Chinese patients with HHT.

## 2 MATERIALS AND METHODS

### 2.1 Ethical compliance

The study was approved by the Ethics Committee of Beijing TongRen Hospital and performed in accordance with the guidelines of the World Medical Association’s Declaration of Helsinki. Written informed consent was obtained from all subjects or from next of kin, and carers or guardians of minors/children.

### 2.2 Cohort

A total of 62 individuals, including 36 females and 26 males, from 31 unrelated families with one or more members suffering from HHT were recruited from the outpatient clinic of Otolaryngology, Head and Neck Surgery Department at Beijing TongRen Hospital, who come from the different provinces in China. All the patients were of Han Chinese origin and aged between 4 years old to 73 years old; with a mean age of 42.9 ± 15.7 years. Clinical diagnosis of HHT was made according to the Curacao criteria (Shovlin et al., 2000). A cohort of 100 individuals without recurrent epistaxis, telangiectasias and the family history of HHT were also recruited as normal controls. Subjects were excluded if they or their first degree family members had any inherited vascular diseases.

### 2.3 DNA extraction

DNA was extracted from the peripheral blood leukocytes using the DNA Isolation Kit (Roche, Indianapolis, USA).

#### 2.3.1 Single nucleotide variants and indel analysis

The protein coding sequences together with intron/exon boundaries of the four related genes (*ENG,* NM_000118.3; *ACVRL1,* NM_000020.2; *SMAD4,* NM_005359.5; *BMP9,*NM_016204.2) were amplified using polymerase chain reaction (PCR) for all DNA samples. The purified PCR products were directly sequenced using BigDye Terminator v.3.1 Cycle sequencing Kit (Applied Biosystems, Foster City, USA) and analyzed on ABI 3,730 DNA Analyzer (Applied Biosystems, Foster City, USA). PCR and sequencing primer pairs were designed using online Primer 3.0 software (Koressaar & Remm, 2007) (Table S1). The coding region and the flanking sequences (about 50 bases around the coding region) of the four genes were captured. Nucleotide alterations were identified by sequence alignment with the NCBI Reference Sequence (Build137). When a novel missense variant was identified, the paralog and ortholog sequences were compared using the CLUSTAL O (1.2.4) Multiple Sequence Alignment Program (Bayrak‐Toydemir, Mao, Lewin, & McDonald, 2004). The functional impact on the protein as an amino acid substitution was assessed using SIFT software (Bayrak‐Toydemir et al., 2006).

### 2.4 Deletion/duplication detection

Large deletions and duplications in the *ACVRL1* and *ENG* genes of individuals who tested negative via PCR amplification and sequencing were detected using the SALSA MLPAkit (P093‐B1 HHT/PPH1, MRC‐Holland, the Netherlands), according to the manufacturer’s instructions. MLPA peak plots were analyzed using the Coffalyser. Net software (MRC‐Holland) to normalize and calculate the dosage ratios. Limit dosage ratios of ≤0.7 and ≥1.35 were set for deletion and duplication, respectively.

Additionally, when a variant likely to be pathogenic was identified in a proband, the variant was screened in other family members to assess whether the variant was co‐segregated with the patients and normal individuals. Furthermore, one hundred unrelated normal individuals were analyzed for each novel variant detected.

### 2.5 Evaluation of variants

The classification for variants uses the joint consensus recommendation of the American College of Medical Genetics and Genomics and the Association for Molecular Pathology. Variants have been classified as pathogenic, likely pathogenic, variant of uncertain significance (VUS), likely benign and benign (Richards et al., 2015). The calculation and analysis for the probability of observed cosegregation was according to the method recommended by Jarvik & Browning (2016).

## 3 RESULTS

Overall, 62 individuals from 31 HHT families were recruited. Among them, five individuals were sporadic with no family history of HHT and the other 57 individuals came from 26 families with other affected members (Figure S1).

Table 1 shows the characteristics of all the participants in the study. Epistaxis was the most frequent clinical feature in our cohort and all the individuals had the manifestation. Overall, 32 patients were diagnosed as definite HHT patients and 11 as possible HHT patients, with the HHT onset age ranging from 3 to 50 years old. Four subjects were classified as “carriers” based on the presence of pathogenic gene variant and the missing symptoms, which may be explained by their rather young age (the age were described in Table 1).

**Table 1:** Clinical features and variant analyses results

Family ID	Individual ID	Gender	Age	Onset age	Epistaxis	MT^a^	AVMs^b^	Family history	Diagnosis	Gene	Exon	Nucleotide change	Amino acid change	Classification
F1	F1_III:1	Male	55	17	Yes	No	GIT	Yes	HHT	*ACVRL1*	EXON8	c.1231C>T	p.Arg411Trp	Pathogenic
F1	F1_III:2	Male	47	18	Yes	Na	Na	Yes	Possible HHT	*ACVRL1*	EXON8	c.1231C>T	p.Arg411Trp	Pathogenic
F1	F1_III:3	Female	40	17	Yes	Na	Na	Yes	Possible HHT	*ACVRL1*	EXON8	c.1231C>T	p.Arg411Trp	Pathogenic
F1	F1_IV:1	Male	28	/	No	No	Na	Yes	Carrier	*ACVRL1*	EXON8	c.1231C>T	p.Arg411Trp	Pathogenic
F2	F2_II:1	Female	58	20	Yes	Yes	PAVMs	Yes	HHT	*ACVRL1*	EXON3	c.200G>A	p.Arg67Gln	Pathogenic
F2	F2_III:1	Male	31	/	No	No	Na	Yes	Normal	/	/	/	/	
F3	F3_II:1	Female	72	18	Yes	Yes	HAVMs	Yes	HHT	***ENG***	**EXON5**	**c.593del**	p.Pro198Argfs*24	**Pathogenic**
F3	F3_III:1	Male	48	20	Yes	Yes	Na	Yes	HHT	***ENG***	**EXON5**	**c.593del**	p.Pro198Argfs*24	**Pathogenic**
F4	F4_II:1	Male	32	26	Yes	Yes	HAVMs	Yes	HHT	*ACVRL1*	INTRON4	c.526−3C>G	/	VUS
F5	F5_I:1	Male	73	23	Yes	Na	HAVMs,PAVMs	Yes	HHT	***ENG***	**EXON7**	**c.841A**>**G**	**p.Ile281Val**	**VUS**
F6	F6_II:2	Male	42	20	Yes	No	Na	Yes	Possible HHT	*ACVRL1*	EXON10	c.1436G>C	p.Arg479Pro	VUS
F6	F6_II:1	Male	44	/	No	No	Na	Yes	Normal	/	/	/	/	
F7	F7_II:1	Female	61	25	Yes	Yes	GIT	Yes	HHT	/	/	/	/	
F7	F7_III:1	Male	43	15	Yes	Yes	Na	Yes	HHT	/	/	/	/	
F7	F7_III:2	Female	50	13	Yes	Yes	Na	Yes	HHT	/	/	/	/	
F7	F7_IV:2	Female	22	13	Yes	Yes	Na	Yes	HHT	/	/	/	/	
F7	F7_III:3	Male	30	/	No	No	Na	Yes	Normal	/	/	/	/	
F7	F7_II:2	Male	58	/	No	No	Na	Yes	Normal	/	/	/	/	
F7	F7_IV:1	Male	32	/	No	No	Na	Yes	Normal	/	/	/	/	
F7	F7_III:4	Female	37	/	No	No	Na	Yes	Normal	/	/	/	/	
F7	F7_II:3	Female	58	/	No	No	Na	Yes	Normal	/	/	/	/	
F8	F8_III:1	Female	35	3	Yes	Yes	Na	Yes	HHT	***ENG***	**EXON7**	**c.840del**	p.Ile281Serfs*78	**Pathogenic**
F8	F8_IV:1	Male	10	3	Yes	No	Na	Yes	Possible HHT	***ENG***	**EXON7**	**c.840del**	p.Ile281Serfs*78	**Pathogenic**
F8	F8_II:1	Male	60	18	Yes	Yes	HAVMs,GIT	Yes	HHT	***ENG***	**EXON7**	**c.840del**	p.Ile281Serfs*78	**Pathogenic**
F9	F9_III:2	Male	41	17	Yes	Yes	Na	Yes	HHT	***ENG***	**EXON14**	**c.1878**+**7C>T**	**/**	**Likely pathologenic**
F9	F9_III:1	Female	43	/	No	No	Na	Yes	Normal	**/**	**/**	**/**	/	/
F9	F9_III:4	Male	40	12	Yes	Yes	Na	Yes	HHT	***ENG***	**EXON14**	**c.1878**+**7C>T**	**/**	**Likely pathologenic**
F9	F9_IV:1	Female	10	4	Yes	No	Na	Yes	Possible HHT	***ENG***	**EXON14**	**c.1878**+**7C>T**	**/**	**Likely pathologenic**
F9	F9_II:1	Female	67	20	Yes	Yes	GIT	Yes	HHT	***ENG***	**EXON14**	**c.1878**+**7C>T**	**/**	**Likely pathologenic**
F9	F9_III:3	Female	39	/	No	No	Na	No	Normal	/	/	/	/	/
F11	F11_II:1	Male	50	10	Yes	Yes	Na	Yes	HHT	/	/	/	/	/
F12	F12_III:1	Male	53	20	Yes	Yes	HAVMs	Yes	HHT	*ACVRL1*	EXON8	c.1232G>A	p.Arg411Gln	Pathogenic
F13	F13_II:1	Female	37	5	Yes	Na	HAVMs	Yes	HHT	/	/	/	/	/
F14	F14_II:1	Female	53	18	Yes	Yes	PAVMs	Yes	HHT	*ENG*	EXON4	c.496del	p.Gln166Argfs*56	Pathogenic
F14	F14_III:1	Female	28	/	No	Na	Na	Yes	Carrier	*ENG*	EXON4	c.496del	p.Gln166Argfs*56	Pathogenic
F14	F14_II:2	Female	50	/	No	Na	Na	Yes	Normal	/	/	/	/	/
F15	F15_II:1	Female	45	17	Yes	Yes	Na	Yes	HHT	*ACVRL1*	EXON7	c.853C>T	p.Leu285Phe	VUS
F15	F15_III:1	Female	10	/	No	No	Na	Yes	Normal	/	/	/	/	/
F16	F16_II:1	Male	57	30	Yes	Yes	HAVMs	Yes	HHT	*ACVRL1*	EXON8	c.1120C>T	p.Arg374Trp	Pathogenic
F16	F16_III:1	Male	23	/	No	No	Na	Yes	Carrier	*ACVRL1*	EXON8	c.1120C>T	p.Arg374Trp	Pathogenic
F18	F18_II:1	Female	33	3	Yes	Na	Na	Yes	Possible HHT	/	/	/	/	/
F18	F18_III:1	Female	11	/	No	No	Na	Yes	Normal	/	/	/	/	/
F19	F19_II:1	Male	57	50	Yes	Yes	Na	Yes	HHT	***ACVRL1***	**EXON7**	**c.1042G**>**A**	**p.Asp348Asn**	**VUS**
F20	F20_III:1	Male	36	28	Yes	Yes	Na	Yes	HHT	***ACVRL1***	**EXON8**	**c.1207C**>**G**	**p.Leu403Val**	**VUS**
F21	F21_III:1	Female	58	15	Yes	Yes	Na	Yes	HHT	*ACVRL1*	EXON8	c.1135G>A	p.Glu379Lys	Pathogenic
F21	F21_III:2	Female	50	14	Yes	Yes	Na	Yes	HHT	*ACVRL1*	EXON8	c.1135G>A	p.Glu379Lys	Pathogenic
F21	F21_IV:1	Female	32	/	No	No	Na	Yes	Normal	/	/	/	/	/
F22	F22_II:1	Female	53	45	Yes	Yes	HAVMs	Yes	HHT	/	/	/	/	/
F23	F23_II:1	Female	41	3	Yes	Yes	Na	Yes	HHT	/	/	/	/	/
F24	F24_II:1	Female	42	14	Yes	Yes	HAVMs	Yes	HHT	*ENG*	EXON6	c.772del	p.Tyr258Thrfs*101	Pathogenic
F25	F25_I:1	Male	45	13	Yes	Yes	Na	Yes	HHT	*ACVRL1*	EXON8	c.1232G>A	p.Arg411Gln	Pathogenic
F25	F25_II:1	Female	4	/	No	No	Na	Yes	Carrier	*ACVRL1*	EXON8	c.1232G>A	p.Arg411Gln	Pathogenic
F26	F26_III:1	Male	45	14	Yes	Yes	Na	Yes	HHT	*ACVRL1*	EXON3	c.200G>A	p.Arg67Gln	Pathogenic
F27	F27_II:1	Female	59	20	Yes	No	Na	Yes	Possible HHT	***ACVRL1***	**EXON5**	**c.576del**	p.Leu193Trpfs*65	Pathogenic
F27	F27_III:1	Female	36	/	No	No	Na	No	Normal	/	/	/	/	/
F27	F27_III:2	Female	33	/	No	No	Na	No	Normal	/	/	/	/	/
F28	F28_III:1	Male	43	13	Yes	No	Na	Yes	Possible HHT	*ENG*	INTRON3	c.360+1G>A	/	Pathogenic
S1	S1	Female	52	45	Yes	Yes	GIT	No	HHT	***ACVRL1***	**EXON5**	**c.552_559delins** **TCTGCTCAGGTGCAGTCT**	p.Gly185Leufs*43	Pathogenic
S2	S2	Female	73	15	Yes	Yes	GIT	No	HHT	*ACVRL1*	EXON8	c.1232G>A	p.Arg411Gln	Pathogenic
S4	S4	Female	63	20	Yes	Yes	Na	No	Possible HHT	*ACVRL1*	EXON8	c.1120C>T	p.Arg374Trp	Pathogenic
S6	S6	Female	31	3	Yes	Yes	Na	No	Possible HHT	*ACVRL1*	EXON3	c.106T>C	p.Cys36Arg	VUS
S8	S8	Female	54	14	Yes	No	PAVMs	No	Possible HHT	/	/	/	/	/

Abbreviations: Na, not available; Yes, the manifestation is present; No, the manifestation is absent; GIT, Gastrointestinal telangiectases; PAVMs, pulmonary arteriovenous malformations; HAVMs, hepatic arteriovenous malformations; Variants in bold red letters are novel.

^a^ Mucocutaneous telangiectasias.

^b^ Arteriovenous malformations.

A total of 20 variants were identified in 24 of 31 kindred (sequences shown in Figure S2), with 24 definite HHT cases from19 kindred, 9 possible HHT individuals from 8 kindred and 4 carriers from 4 kindred, which were responsible for 77.4% (24/31) of all HHT families. No variant was detected in 7 families (7/31, 22.6%), including 5 families with 8 definite HHT patients and 2 families with 2 possible HHT patients (Table 1).

All the 20 variants were single nucleotide variants (SNVs) or small indels located in *ACVRL1* and *ENG* gene. We didn’t find any pathogenic variant in the *SMAD4* or *BMP9* gene. No gross alteration was found in the MLPA analysis for *ACVRL1* and *ENG*.

**Figure 1:**
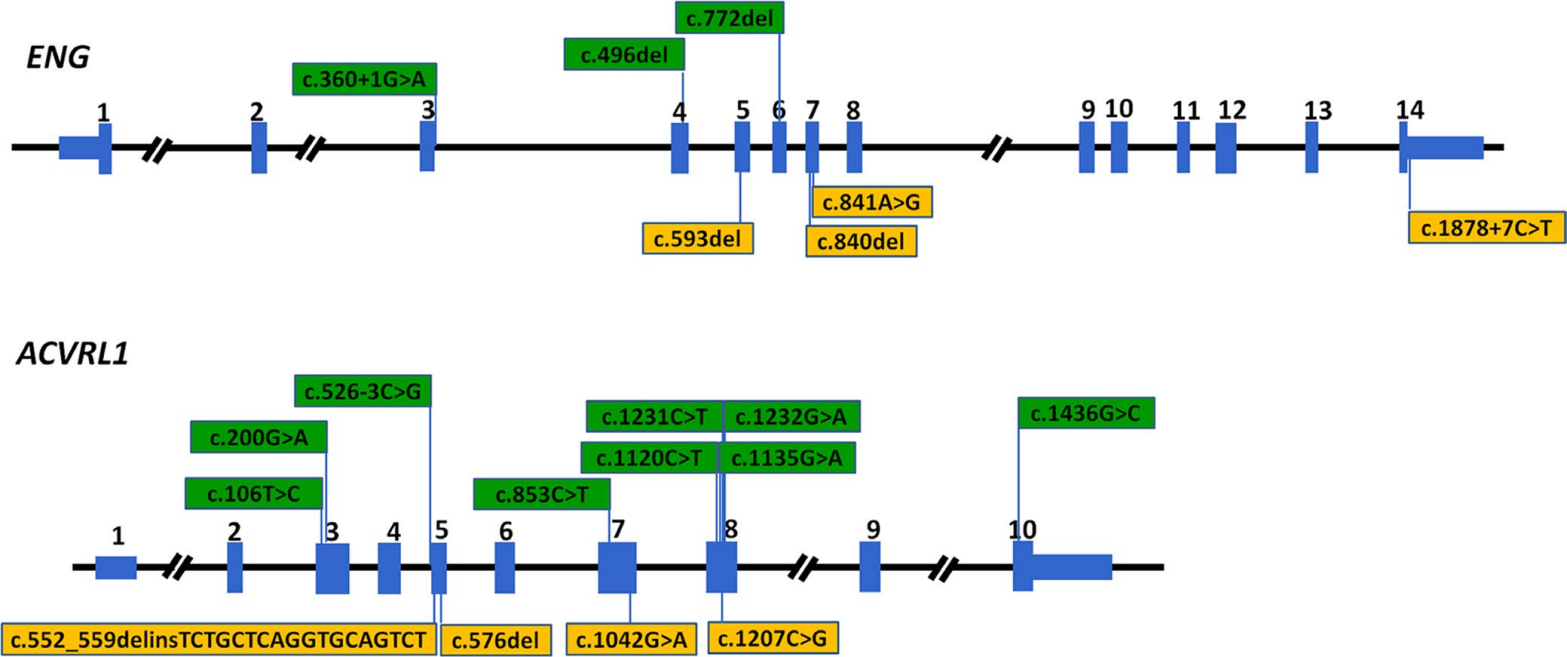
Variants found in the HHT families and their sketches on the *ENG* and *ACVRL1* genes. The novel variant was shown as orange bars, and the reported variants are shown as green bars

A total of 13 variants in *ACVRL1* gene were detected from 17 isolated HHT families (17/24 families, 70.8%). The *ACVRL1* variant of c.200G>A in exon3, the c.1120C>T and c.1232G>A in exon8 were recurrent in unrelated families. Overall, variants found in 8/17 (47.1%) of all families with *ACVRL1* variants were located in exon8 (including 5 unique variants) (Figure 1). The distribution of other *ACVRL1* variants was illustrated on Figure 1. Similarly, seven variants in *ENG* gene were identified in 7 HHT families (7/24 families, 29.2%). The distribution of the seven *ENG* variants was showed on Figure 1.

A total of eight novel variants, which have not been reported previously, were found in this study. Four of these were detected in the *ENG* gene (c.593del, c.840del and c.1878+7C>T and c.841A>G), and the other four novel variants were located in the *ACVRL1* gene (c.576del, c.1207C>G, and c.552_559delinsTCTGCTCAGGTGCAGTCT and c.1042G>A). We further analyzed the pathogenic potential for the different type of novel variants in the following section.

Four of the novel variants were out‐of‐frame indels, which may be pathogenic as haploinsufficiency of *ENG* or *ACVRL1* was an underlying cause of HHT (Pece‐Barbara, Cymerman, Vera, Marchuk, & Letarte, 1999). These variants have never been found in public databases (dbSNPs, 1000Genome and ESP) neither the previous investigations. Patient’s phenotype is highly specific for HHT. Variant of c.552_559delinsTCTGCTCAGGTGCAGTCT in *ACVRL1* was found in a sporadic individual. In regards to c.593del, c.840del in *ENG* and the c.576del in *ACVRL1,* variants testing for the family members found cosegregation with disease in more than one family member*.* Variant of c.593del was found in a four generation family (family ID: F3, Figure S1). Both the proband and her son, who were clinically diagnosed as definite HHT, were heterozygous with the variant. Samples of the other family members were not obtained. The second out‐of‐frame variant (c.840del) was detected in a four generation family with three patients. The proband, her father and her son were heterozygous with variant of c.840del (family ID: F8, Figure S1). The proband and her father were clinically diagnosed as HHT with more than three features. And the proband’s son, a possible HHT individual, was confirmed by the genetic testing. The novel c.576del was found in a four generation family (family ID: F27, Figure S1). In this family, a HHT patient and two normal individuals were recruited. The HHT patient was heterozygous with the c.576del variant and the two normal individuals were wildtype. And the genotype for all obtained members in F27 was co‐segregated with the manifestation of the HHT. All the above findings indicated that these novel variants were likely to be pathogenic.

The fifth novel variant (c.1878+7C>T,) was a substitution in the 3′UTR region of the *ENG*. It was found in a four generation family (family ID: F9, Figure S1). In this family, 6 DNA samples from 4 patients and 2 normal individuals were obtained and tested. All 4 patients were heterozygous with this variant of c.1878+7C>T, and the 2 normal individuals were wild type for this site. The variant was rare and not found in the previous reports and the public databases (dbSNPs, 1000Genome and ESP), thus, we assumed the variant only entered the pedigree once. The probability of observed cosegregation was calculated. The untyped relative (II:2) who must had passed the variant was assumed to be heterozygous. Considering definitely affected individuals (Ⅱ:3, Ⅲ:3, Ⅲ:5 and Ⅳ:3), we observed four meioses, so the affected individuals contributed a factor of (1/2)^4^ to the value of probability of observed cosegregation. The normal individual (Ⅲ:1) from the family contributes a factor of (1−(1/2)) = 1/2. Thus, for family F9, the probability of observed cosegregation was 1/32, which is a strong evidence for the pathogenicity of c.1878+7C>T in the *ACVRL1* (Jarvik & Browning, 2016).

The other three novel missense variants (c.841A>G in *ENG*, c.1207C>G and c.1042G>A in *ACVRL1*) were absent in the 100 normal individuals. Variants of c.1207C>G and c.1042G>A were not found in the public databases (dbSNPs, 1000Genome and ESP) and the previous studies. This variant of c.841A>G was reported in the EXAC (The Exome Aggregation Consortium) database (Cymerman, Vera, Karabegovic, Abdalla, & Letarte, 2003). The Minor allele frequency of this variant was 1.7e‐5 in the dbSNPs database. SIFT was applied to predict whether the missense variants affect protein function. It was predicted to be “damaging”. Alignment for amino acid sequences from different species found that the novel missense variants were located in the conserved region (Figures S3 and S4).

## 4 DISCUSSION

HHT presents clinically as a variety of symptoms including recurrent epistaxis, mucocutaneous telangiectasias, and visceral AVMs in lung, liver, gastrointestinal tract, brain or spinal cord. In the present study, most of the patients diagnosed as definite HHT patients demonstrated epistaxis, mucocutaneous telangiectasia and family history. Nose bleeds were observed in all definite individuals, but also the 11 possible HHT individuals.

Our study has demonstrated that 77.4% of the kindred (24/31, 24 families including 24 definite HHT cases, 9 possible HHT cases, 4 carriers) had *ACVRL1* or *ENG* variant, which is in accordance with the findings of previous reports (Bayrak‐Toydemir et al., 2006; Chen et al., 2013; Heimdal et al., 2016). However, the variant carrier percent in HHT from the ARUP institute is higher than that of our study, as the non‐coding region variants are screened, which may contribute to about 1% of HHT patients (Wooderchak‐Donahue et al., 2018). What’s more, none variant was found in *SMAD4* and *BMP9* gene, which may account for about 1%–2% of HHT patients. In the present study, a likely or clearly pathogenic variant was detected in 7 possible HHT cases and 4 individuals without any symptoms except for positive family history, which enabled us to set the diagnosis of HHT for the recurrent epistaxis patients and find the high risk individuals early. These findings suggest that the Curacao Criteria should be revised to take into consideration the results of genetic testing, which could confirm the recurrent nose bleeds and find the high risk individuals early (Torring, Brusgaard, Ousager, Andersen, & Kjeldsen, 2014).

Previous studies have indicated that there is a considerable variation in the *ENG*/*ACVRL1* variant ratio in different populations. While one study in French HHT patients has demonstrated the *ENG*/*ACVRL1* variant ratio range from 0.37 to 0.51 (Lesca et al., 2007), a *ENG*/*ACVRL1* variant ratio of 0.72 (13/18) has been found in Canadian patients (Abdalla et al., 2005), a ratio of 1.22 in American (USA) patients (Bayrak‐Toydemir et al., 2006) and a ratio of 2.0 in Danish patients (Brusgaard et al., 2004). In the current study, 17 families (17/24, 70.8%) had variants in *ACVRL1* gene and 7 families (7/24, 29.2%) in the *ENG*, providing an *ENG*/*ACVRL1* variant ratio of 0.41 (7/17), which is comparable to the findings in the French patients. In comparison, another study of patients from 14 Chinese families has indicated a ratio is 0.25 (2/8) (Chen et al., 2013). It is possible that the wide variation noted in the *ENG*/*ACVRL1* variant ratios in different populations may be a consequence of differences in patient numbers and study methods employed in the different studies. However despite these differences, the findings from the two studies of Chinese HHT patients indicate that *ACVRL1* variants, which were 2.4–4 times greater than *ENG* variants, are the predominant cause of HHT in the Chinese patients.

In *ACVRL1*, the c.1120C>T (p.Arg374Trp) and c.1232G>A (p.Arg411Gln) variants on exon8 were seen twice and three times in apparently unrelated families. These two variants have been reported in several families in previous studies (Abdalla, Cymerman, Johnson, Deber, & Letarte, 2003; Bayrak‐Toydemir et al., 2006; Berg et al., 1997; Harrison et al., 2003; Johnson et al., 1996; Kjeldsen et al., 2001; Lesca et al., 2004; Trembath et al., 2001), suggesting that these codons may be the hotspot or founder region of the *ACVRL1* gene, which may need further studies in a larger sample size to proved. In this study, variants in 8/17(47.1%) families were located in exon8 of *ACVRL1* gene. Indeed, a study by Chen and colleagues (Chen et al., 2013) investigating 14 Chinese HHT families also reported 8 unique *ACVRL1* variants, of which 3/8 (37.5%) variants (c. 1121G>A, c.1124A>G and c.1195T>C) were located in exon8. These findings suggest that exon8 of the *ACVRL1* gene may be a hotspot region, which may be useful in the effective genetic testing for HHT. In contrast, seven of the *ENG* variants were widely distributed throughout the gene, none of which was observed in multiple families.

In this study, a total of eight novel variants were found and the pathogenicity was evaluated. Four of the novel out‐of‐frame indels were proved to be pathogenic for HHT. The variant of c.1878+7C>T in *ENG* was found in a four generation family. The cosegregation with HHT in this family was a strong evidence for the pathogenicity of c.1878+7C>T. Besides that, the variant was rare and absent in the public databases. These results strongly supported that it was to be “likely pathogenic”, although all variants in the two terminal exons except a large deletion (two exons) were currently listed as benign or pending classification in ARUP ([Link]; Jarvik & Browning, 2016; Richards et al., 2015). The three missense variants were absent or rare in the public database. Multiple lines of computational evidence support a deleterious effect on the gene. However, the functional study for the pathogenicity of these variants is required in the future studies. So, the three missense variants were classified as “variant of unknown significance (VUS)” according to standards and guidelines of the American College of Medical Genetics and Genomics (Richards et al., 2015).

Indeed, a total of 12 variants have ever been reported in the previous studies, including two out‐of‐frame indels (c.496del([Link]; Lesca et al., 2004) and c.772del(Fernandez et al., 2006; Olivieri et al., 2007)), two assumed splice‐site variants (c.526‐3C>G(Torring et al., 2014) and c.360+1G>A(Cymerman et al., 2003, 2000; Pece et al., 1997)) and eight missense variants (c.1231C>T(Abdalla, Geisthoff, et al., 2003; Trembath et al., 2001; Zhang et al., 2004), c.200G>A(Bayrak‐Toydemir et al., 2004; Berg et al., 1997; Olivieri et al., 2007; Schulte et al., 2005), c.1232G>A(Abdalla, Geisthoff, et al., 2003; Bayrak‐Toydemir et al., 2004; Berg et al., 1997; Johnson et al., 1996), c.1120C>T(Abdalla, Cymerman, et al., 2003; Berg et al., 1997; Kjeldsen et al., 2001), c.1135G>A(Bayrak‐Toydemir et al., 2004; Brusgaard et al., 2004; Lesca et al., 2004), c.1436G>C(Bayrak‐Toydemir et al., 2006; Lesca et al., 2006), c.853C>T(Bayrak‐Toydemir et al., 2004; Lesca et al., 2004; J. McDonald et al., 2011) and c.106T>C([Link])). The two out‐of frame indels located in *ENG* have been proved to be pathogenic. Variant of c.360+1G>A located in the invariant splice‐site sequence GU (+1+2) or AG (−1 –2), and expected to cause aberrant splicing. Five of the eight missense variants (c.1231C>T, c.200G>A, c.1232G>A, c.1120C>T and c.1135G>A) have been reported to be pathogenic in the previous studies (Bossler, Richards, George, Godmilow, & Ganguly, 2006). The other three (c.1436G>C, c.853C>T and c.106T>C) were found to be all located in the conserved region aligning with amino acid sequences from different species (Figure S3), suggesting potential pathogenic role in HHT. However, it may need more information to support this.

In conclusion, our study has demonstrated that 24 of the 31 (77.4%) kindred carry the variant from *ACVRL1* or *ENG* gene. And no variant to be likely pathogenic in *SMAD4* and *BMP9* was found. HHT patients with *ACVRL1* variants are 2.4–4 times more than those with *ENG* variants in Chinese. About 47.1% of the *ACVRL1* variants are located in exon8, despite a wide distribution throughout the gene. These findings suggest that exon8 of the *ACVRL1* gene may be a hotspot region for HHT in Chinese patients. And c.1120C>T (p.Arg374Trp) and c.1232G>A (p.Arg411Gln) in *ACVRL1* may be the commonest variants. Further studies with a larger sample size and functional analysis for the variants are needed to confirm this.

## CONFLICT OF INTEREST

The author(s) declared no potential conflicts of interest with respect to the research, authorship, and/or publication of this article.
