# Supplementary material for: Variant analysis in Chinese families with hereditary hemorrhagic telangiectasia
Source: Mol Genet Genomic Med. 2019 Aug 10;7(9):e893. doi: 10.1002/mgg3.893 (PMC6732279; doi:10.1002/mgg3.893)

**Supplementary TABLE 1** Primers used in the PCR and sequencing for the coding region of *ACVRL1*, *ENG*, *BMP9* and *SMAD4*

| **Primers** | **Exon** | **Forward (5'to 3')** | **Reverse (5'to 3')** | **L*ENG*th** |
| --- | --- | --- | --- | --- |
| *ACVRL1*-2 | exon2 | ACATTGCTCTCCACCCTTCA/ | CAGCTTCTCAAGTTCAGCCC | 392 |
| *ACVRL1*-3 | exon3 | AGACGAGAGGGACAGTAGGA/ | AAGAAGATGGGGAGGGAGTG | 382 |
| *ACVRL1*-4 | exon4 | GACTCTGGGATCTAACTGGCA | CGGCTCTAATCTCTGGGTGA | 389 |
| *ACVRL1*-5 | exon5 | GAGTGAGGAGCTTGCAGTGA | ACCGCCTGTGATTCCAGTAG | 295 |
| *ACVRL1*-6 | exon6 | ACTGGGTTTGGGTCTGGATT | GAGGTCTGCAAACTTGAGCC | 372 |
| *ACVRL1*-7 | exon7 | GACCCAGTCCATTCCCTCTC | GTGCTAATCATGGTCACCGC | 475 |
| *ACVRL1*-8 | exon8 | CCCTCTCTGTCCCACTGTTT | TCTGACTGCAAACCTCCCAG | 400 |
| *ACVRL1*-9 | exon9 | AGAGGGTAGAAAAGGCTCTCC | GCCTCAGACACAAGTTCCTG | 298 |
| *ACVRL1*-10 | exon10 | GGCCATCCTCCTCATCTTCT | CTCTTTTGCATCCTGTCCCG | 556 |
| *ENG*-1 | exon1 | GCACTTCCTCTACCCGGTT | CCCGAGGCTTTCTTTCAACA | 687 |
| *ENG*-2 | exon2 | TGTGATGATGCAGGAAAGCC | TAACGAGGACTCAGCCACTG | 382 |
| *ENG*-3 | exon3 | GGAGAGTGGAGTGGAAGCAT | AGATGAAAGGGAGAAGCAGGG | 362 |
| *ENG*-4 | exon4 | CAATGGGCTGACTCCACAAA | TTGTGGCATGTGAACTGTGG | 396 |
| *ENG*-5 | exon5 | CCACTATCTTTGGCTGTGGG | GGGCTTTATAAGGGACCGGA | 347 |
| *ENG*-6 | exon6 | CCTATCCCATAAACCCACACCT | TGATTTGTCCTTCAGCTCAGC | 354 |
| *ENG*-7 | exon7 | ACCTATGCCCATACGTGAGG | CTCCCATTGTTCCCATGTGC | 476 |
| *ENG*-8 | exon8 | AGAGCCTGAGAATCGCTTGA | AACTAAGGCTTGCAGAGGGA | 456 |
| *ENG*-9 | exon9 | GAATGGCTGTGACTTGGGAC | CTCTCCCAAACACACCTCCA | 396 |
| *ENG*-10 | exon10 | AAAATGGGCGTATTGGGTGG | GGCATTCCAGACACACATGG | 241 |
| *ENG*-11 | exon11 | CATGATGCCTGTTCCTCCC | TGGAGTCATGGTGGGAAGAA | 379 |
| *ENG*-12 | exon12 | CTCAGGGGTGGGAACTCTTA | CCATGTCCCTTCCTGCAAA | 434 |
| *ENG*-13 | exon13 | TGGAGATGGGATTCAAAGCC | AGCCAATAACTGTGGGGATG | 460 |
| *ENG*-14 | exon14 | CTGTGATGAGCCCGTTTGC | CCACTGGGTTGAAGGTTCTG | 755 |
| *BMP9*-1 | exon1 | CATGCCCTGTGTGTTTGTCA | AGCCACGCATTTGAAAGGAA | 799 |
| *BMP9*-2 | exon2 | ACTTTAAGGGCTTGGGTGAAAC | GTGCAATGATCCAGCTGTCC | 845 |
| *BMP9*-2-1 | exon2 | CATGAACAAGAGAGCGTGCT | CTTGCATCCCAACAACCCTC | 700 |
| *BMP9*-2-2 | exon2 | GAGTGTGGGTGCAGGTAGTA | AAGATCCTGGGCTTTGGTGT | 586 |
| *SMAD4*-2 | exon2 | CCAGAGCAATTTCATCTTTTCCC | ACCCTGTAGTAGCTTGAAAGGA | 547 |
| *SMAD4*-3 | exon3 | TGAGTTGGTAGGATTGTGAGGA | CGCGGGCTATCTTCCAAATT | 369 |
| *SMAD4*-4 | exon4 | AATTTGGAAGATAGCCCGCG | CTGCCGCTCACACAAACTAA | 472 |
| *SMAD4*-5 | exon5 | GCTGTTACCGCTGAATAAATGAC | TGGGAGCTTAAGAGGCTACTTA | 670 |
| *SMAD4*-6 | exon6-7 | CTGATAGGCCATGGGTGAGT | ACAGAAAACAAAGCCCTACCAA | 539 |
| *SMAD4*-8 | exon8 | GCTGTGTGACCATTGACAAGT | TGTGCGTTTCAATCACCACT | 680 |
| *SMAD4*-9 | exon9 | ATCTCCCCTCCCTTTACCCT | ACCGACAATTAAGATGGAGTGC | 465 |
| *SMAD4*-10 | exon10 | ACTACATGCTCCTGACACATAGT | TTCCATTCCTTCCACCCAGA | 479 |
| *SMAD4*-11 | exon11 | TCCAAGCCACCTTTCCTAACT | CCCCTTTCTCCTTCATCCCA | 557 |
| *SMAD4*-12 | exon12 | CCTTAACCAAAAGTGTGCAGC | TCCAGTTTCTGTCTGCTAGGA | 562 |

**Supplementary Figure1.** Pedigree of all the HHT families. Filled symbols for males (squares) and females (circles) represent affected individuals, and empty, unaffected ones. An arrow denotes the proband. A symbol with dot indicates the mutation carrier who does not present any manifestation.


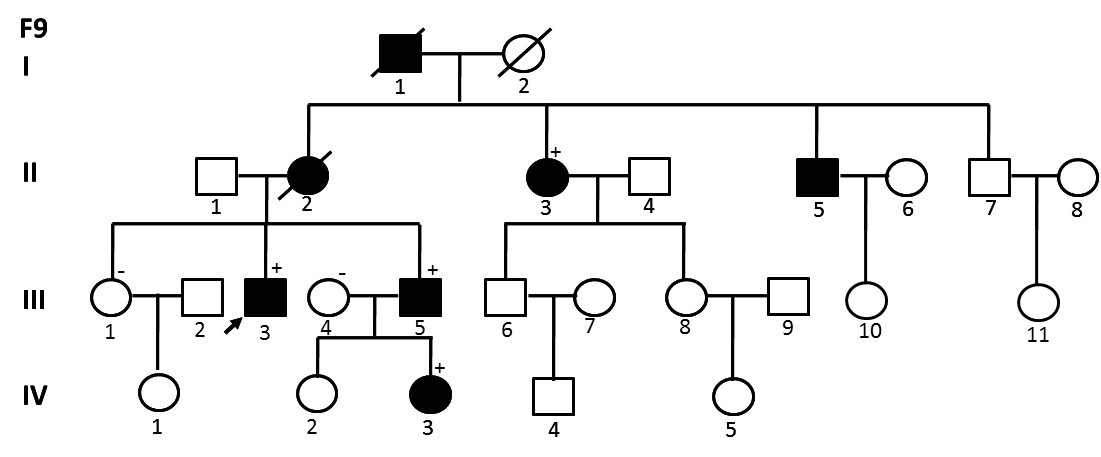


**Supplementary Figure 2.** Sequncing chromatograms of mutations found in the HHT families. The lower panel of each picture showed the heterozygous substitution in affected individuals compared with that of normal control (upper panel).


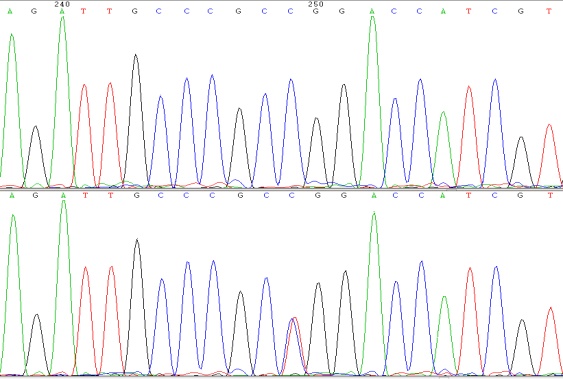

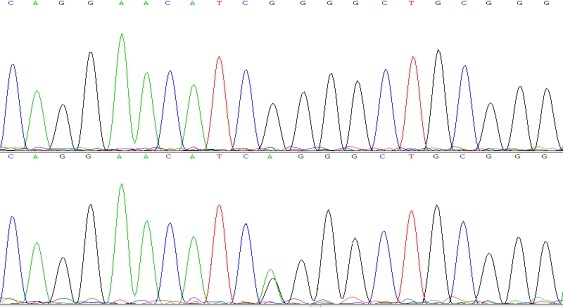


F1: *ACVRL1*: c.1231C>T(p.Arg411Trp) F2: *ACVRL1*: 1c.200G>A (p.Arg67Gln)


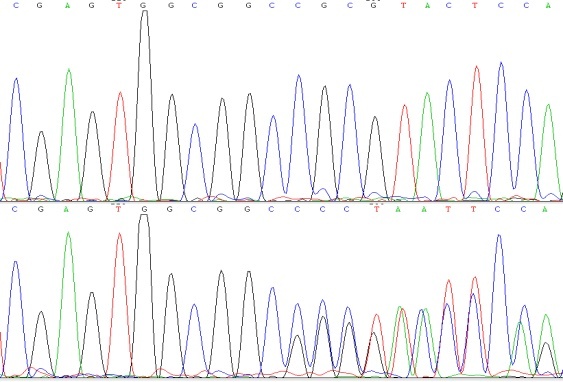

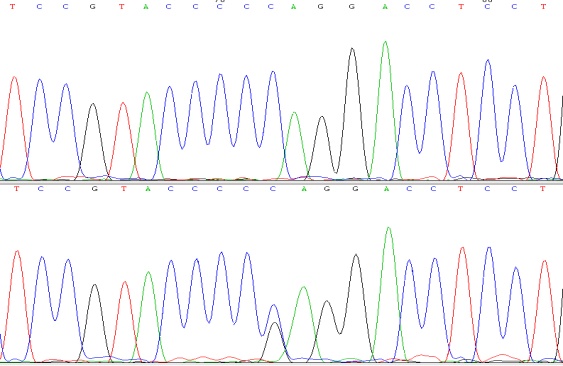


F3: *ENG*: c.593del (p.Pro198Argfs^*^24) F4: *ACVRL1*: c.526-3C>G


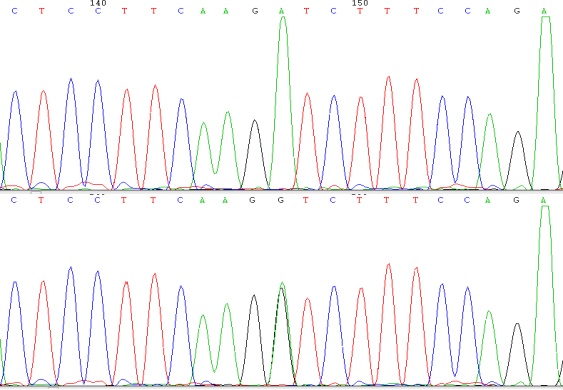

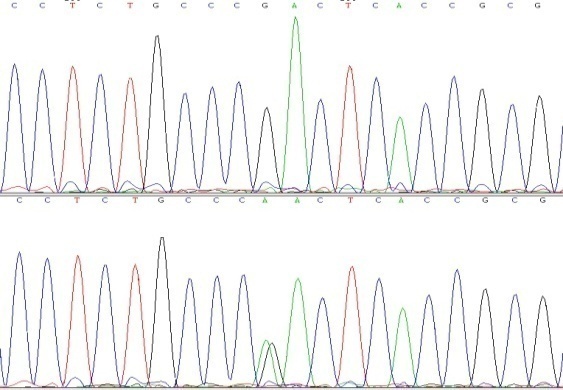


F5: *ENG*: c.841A>G (p.Ile281Val) F6: *ACVRL1*: c.1436G>A (p.Arg479Pro)


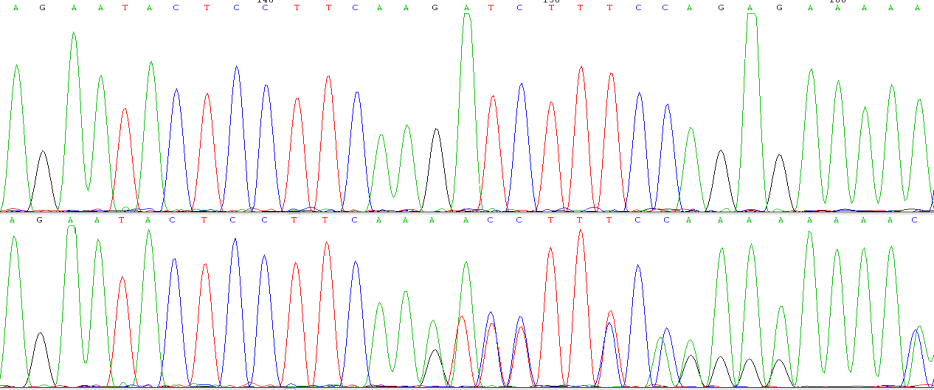


F8: *ENG*: c.840del (p.Ile281Serfs^*^78)


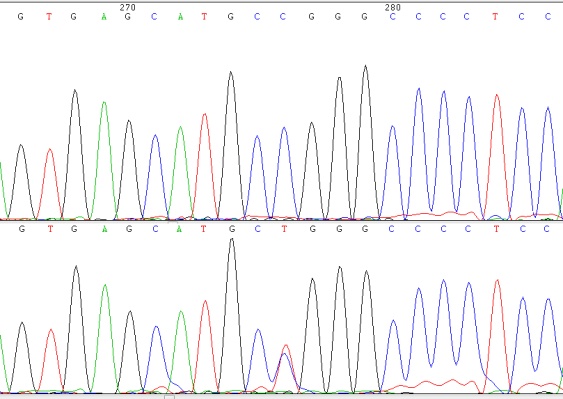

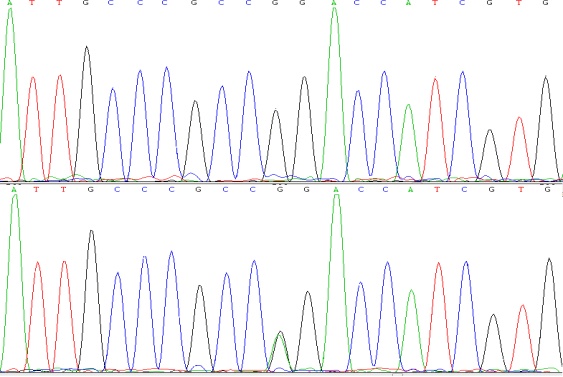


F9: *ENG*: c.1878+7C>T F12: *ACVRL1*: c.1232G>A (p.Arg411Gln)


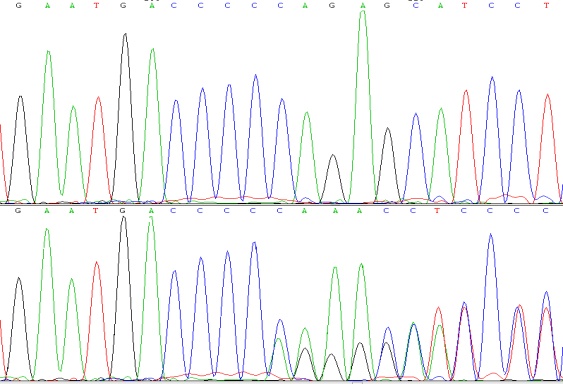

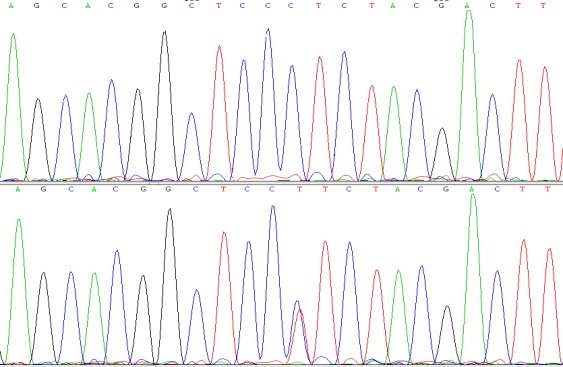


F14: *ENG*: c.496del (p.Gln166Argfs*56) F15: *ACVRL1*: c.853C>T (p.Leu285Phe)


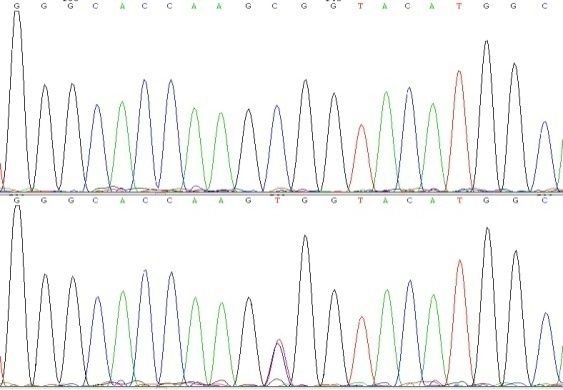

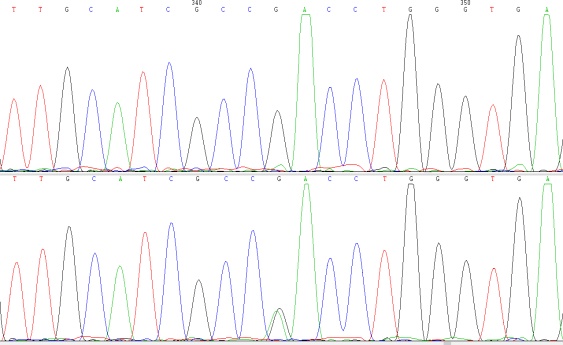


F16: *ACVRL1*: c.1120C>T (p.Arg374Trp) F19: *ACVRL1*: c.1042G>A (p.Asp348Asn)


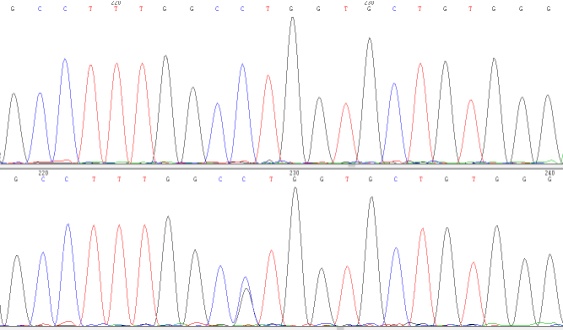

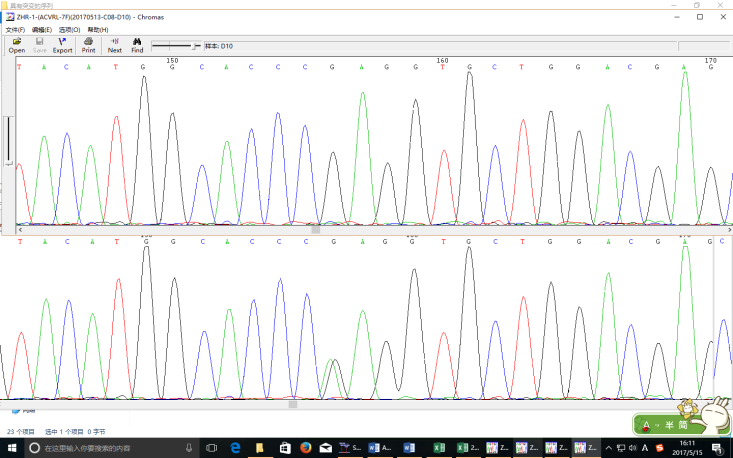


F20: *ACVRL1*: c.1207C>G (p.Leu403Val) F21: *ACVRL1*: c.1135G>A (p.Glu379Lys)


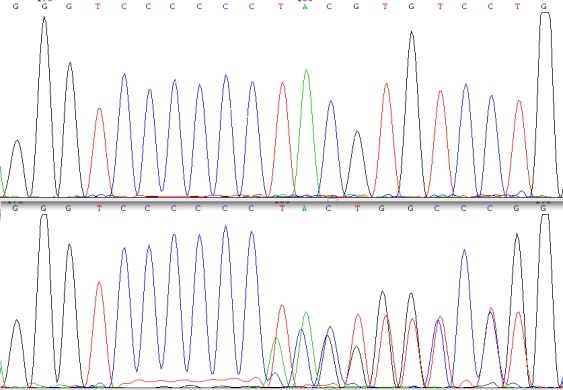

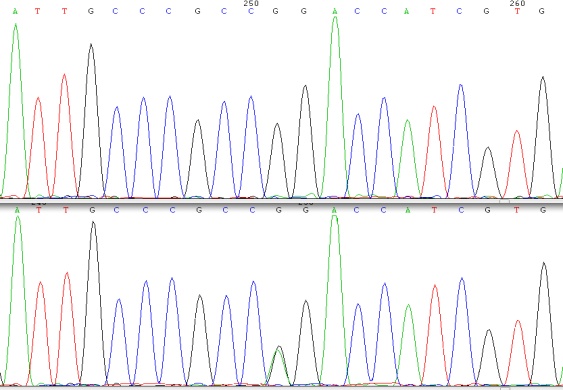


F24 *ENG*: c.772del (p.Tyr258Thrfs*101) F25 *ACVRL1*: c.1232G>A (p.Arg411Gln)


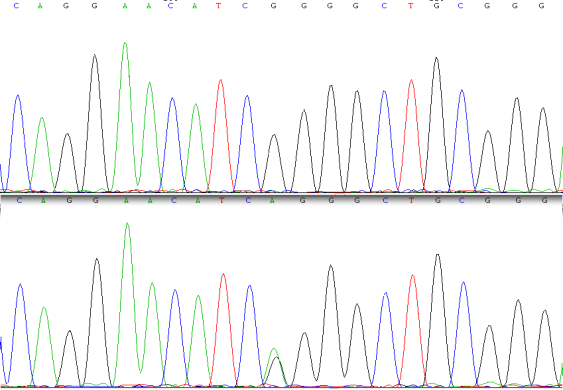

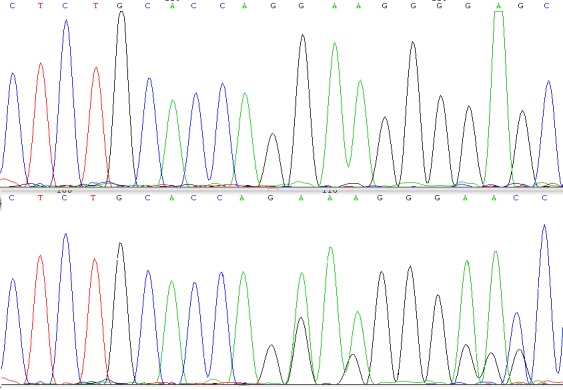


F26 *ACVRL1*: c.200G>A (p.Arg67Gln) F27 *ACVRL1*: c.576del (p.Leu193Trpfs*65)

Reverse complement sequence


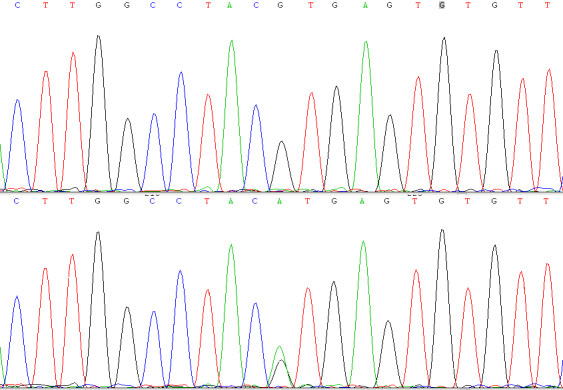


F28 *ENG*: c.360+1G>A


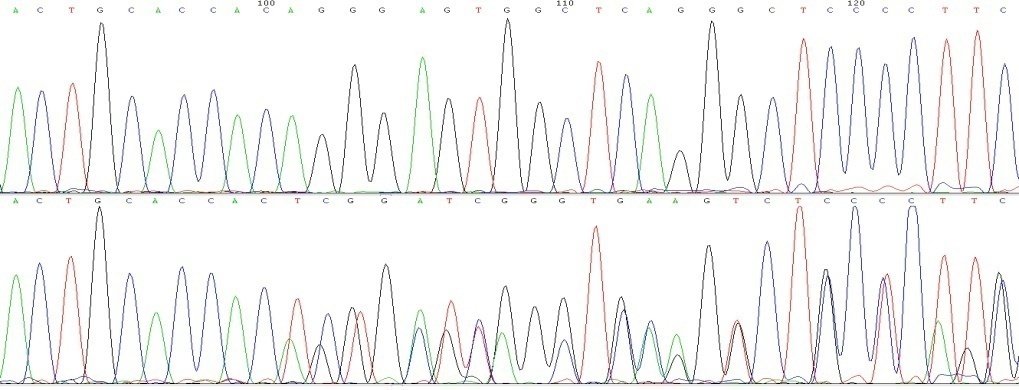


S1: *ACVRL1*: c.552_559delinsTC TGCTCAGGTGCAGTCT (p.Gly185Leufs*43)


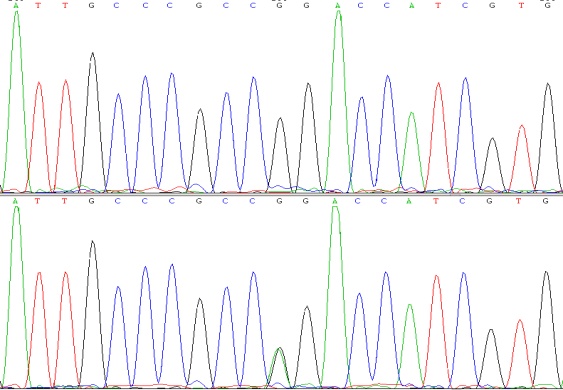

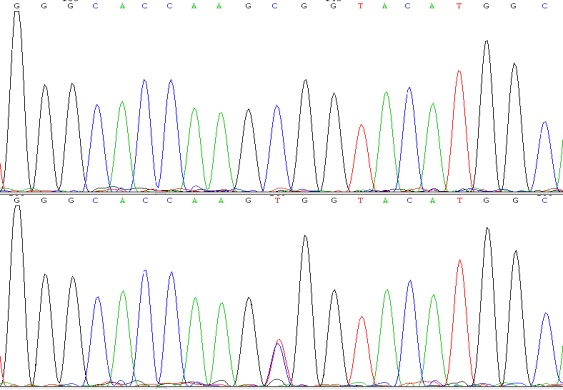


S2: *ACVRL1*: c.1232G>A (p.Arg411Gln) S4: *ACVRL1*: c.1120C>T (p.Arg374Trp)


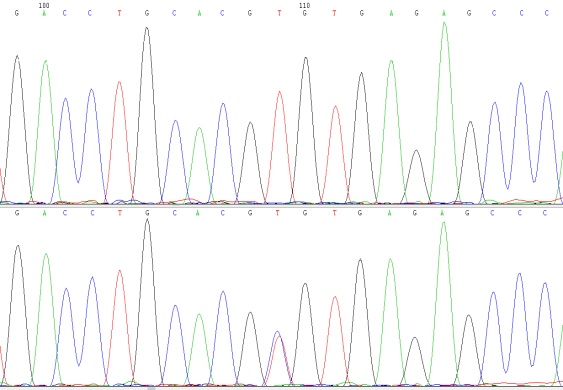


S6: *ACVRL1*: c.106T>C (p.Cys36Arg)

**Supplementary Figure 3.** An alignment of *ACVRL1* from different species (prepared using CLUSTAL O(1.2.4)). All substituted amino acids are fully conserved between species.


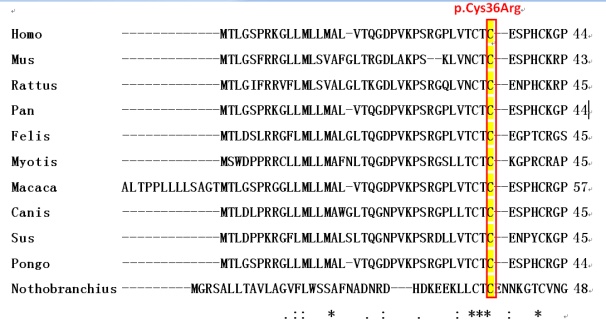

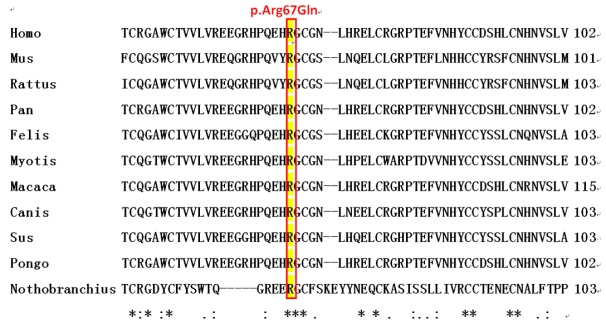


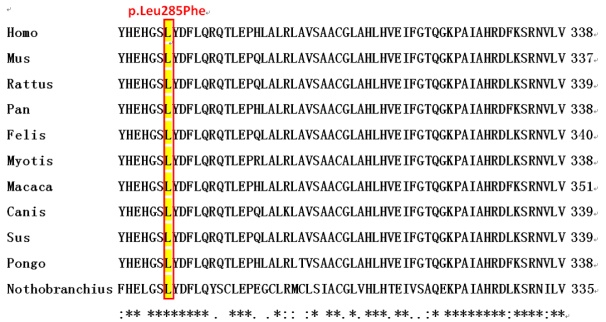

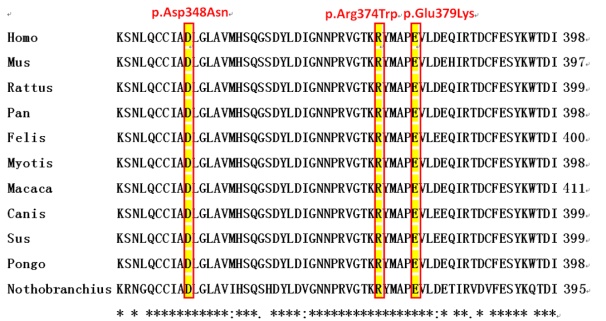


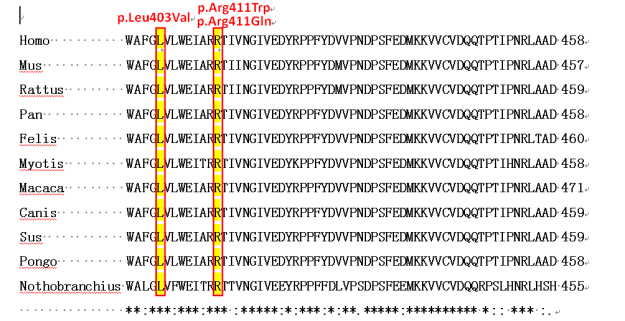

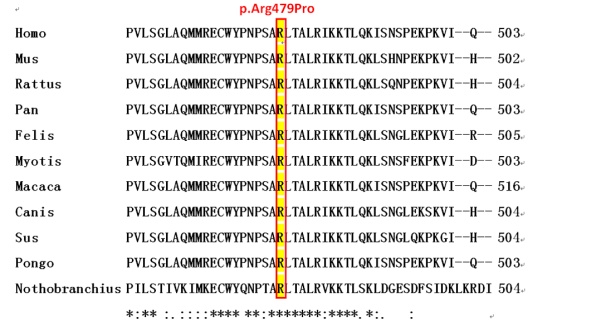


**Supplementary Figure 4.** An alignment of *ENG* from different species (prepared using CLUSTAL O(1.2.4)). The substituted amino acid of p.Ile281Val in the protein kinase domain is conserved between species.


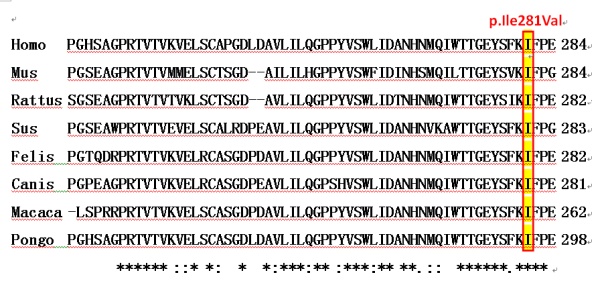

Supplement: Supplementary file 1 [file MGG3-7-e893-s001.docx]
